# Editorial: (Un)healthy lifestyles, aging, and type 2 diabetes

**DOI:** 10.3389/fragi.2025.1696856

**Published:** 2025-12-11

**Authors:** Pedro Duarte-Mendes, Fernanda M. Silva, Ana M. Teixeira

**Affiliations:** 1 Polytechnic Institute of Castelo Branco, School of Education, Castelo Branco, Portugal; 2 Sport Physical Activity and Health Research & Innovation Center (SPRINT), Castelo Branco, Portugal; 3 Faculty of Sport Sciences and Physical Education, CIPER ‐ Interdisdiplinary Center for the Study of Human Performance, University of Coimbra, Coimbra, Portugal; 4 School of Education and Communication, University of Algarve, Faro, Portugal

**Keywords:** sedentary time, exercise, insulin resistance, metabolic syndrome, healthy aging

Recent estimates from the IDF Diabetes Atlas indicate that diabetes affects 589 million adults worldwide in 2024, with type 2 diabetes (T2D) accounting for more than 90% of cases (International Diabetes Federation, 2025). This number is projected to reach 853 million by 2050. Age is an important risk factor for T2D, with estimates indicating that one in four adults with the disease (158 million) are over 65 years old.

Poor lifestyle factors, such as lack of exercise, sedentary behavior, and poor diet, are important underlying factors in the development of obesity and insulin resistance, leading to T2D and cardiovascular disease (Lu et al., 2024; Bowden Davies et al., 2018). On the other hand, effective interventions, such as regular exercise and a healthy diet, have been recommended to improve glucose metabolism in both diabetic and non-diabetic individuals (Silva et al., 2024; Syeda et al., 2023; Kim and Kwon, 2024). In addition to engaging in regular exercise, studies have demonstrated the benefits of interrupting sitting time with regular bouts of movement (Syeda et al., 2023). Given the high prevalence of T2D, mainly in the middle-aged and elderly population, it is important to investigate the impact of (un)healthy lifestyles on metabolic markers and to create a set of strategies to prevent or control T2D and its related complications. This Research Topic, *Editorial: (Un)healthy lifestyles, Aging, and Type 2 Diabetes,* addresses these questions through nine complementary contributions.

The opening article by Wang et al. focuses on elderly health management services, one of China’s basic public health projects, along with the psychological care program for older adults in Shenzhen, Guangdong Province. This is highly relevant considering that China has one of the largest elderly populations in the world, with more than 264 million people aged 60 or older. Using the Long-gang District’s efforts to integrate physical and mental health services as an example, this review highlights the challenges of elderly health work and the potential for combining mental and physical health in medical care. The authors emphasize the importance of prioritizing elderly needs, reinforcing community health services, establishing integrated teams, enhancing public awareness and health literacy, strengthening collaboration across system levels, and improving coordination between healthcare and social support services to build a stronger public health system for the elderly and advance the broader goal of Healthy China.

The study by Zhang et al. aimed to assess the global, regional, and national burden of T2D attributable to low physical activity from 1990 to 2021 and predict its global burden by 2050. Utilizing data from the Global Burden of Disease 2021 data, authors estimated that T2D attributed to low physical activity caused approximately 149,214 deaths and 5.5 million disability-adjusted life-years (DALYs), with an increase since 1990. Both age-standardized mortality and disability-adjusted life years increased, particularly among women. The highest incidence occurred in the elderly (70–74 and 95+ years old). High sociodemographic index (SDI) regions report the highest mortality rates, with rapid growth in areas with high-middle SDI. Projections from 2022 to 2050 indicate continued growth in deaths and DALYs.

The study by Jiang et al. used bibliometric analysis to comprehensively and systematically organize the literature related to the modulation of gut microbiome for the treatment of T2D and to provide guidance for future studies. The authors found that current research focuses mainly on the effects of diet, exercise, and pharmacological modification of the gut microbiome to improve T2D and explores the mechanisms involved in this process. They also stated that future research will delve more deeply into the specific mechanisms that regulate gut microbiome to ameliorate T2D and will extend to T2D-related diseases and symptoms. In this sense, it will be essential to develop new experimental techniques and assays to advance this field.

Cognitive function and its association with T2D are the focus of two studies. The cohort study by Wei and He investigated the potential mediating role of cognitive impairment on the relationship between T2D and all-cause mortality among 1,891 elderly individuals from the National Health and Nutrition Examination Survey (NHANES) database. The findings revealed that older adults with T2D had a significantly higher likelihood of experiencing cognitive impairment and that T2D was associated with an increased all-cause mortality. Moreover, results suggested a mediating role of cognitive impairment in the association between T2D and all-cause mortality, with a mediation effect of 16.2%. The study by Srikantha et al. examined the relationship between T2D and cognitive function among older adults in India and China. Cross-sectional analysis showed no association between T2D and cognition in fully adjusted models for both countries. However, the area of residence and level of education modified these relationships. In India, T2D was positively associated with cognition in rural areas and among individuals with none or early childhood education, with no relationship among those with at least an upper secondary education. In China, an inverse association was observed only among those with less than lower secondary education, with no association among the remainder of the sample. According to the authors, these results differ from those observed in high-income countries, which may indicate ongoing effects of the epidemiological and nutritional transitions observed in India and China.

The study by Bai et al. investigated the prevalence of physical, psychological, and cognitive comorbidities among older adults with diabetes in rural areas and examined the impact of lifestyle factors on health outcomes across different co-morbidity conditions. Authors found a relatively high prevalence (86.08%) of physical, psychological and cognitive co-morbidity among participants. Factors such as regular physical activity, adequate sleep, healthy diet and active social participation were correlated with a lower prevalence of co-morbidity. The authors reinforce the importance of multifactorial lifestyle interventions in diabetic individuals to reduce the risk and burden of co-occurring conditions.

The qualitative study by Skoradal et al. aimed to explore the motivational factors influencing physical activity engagement among individuals with T2D in the Faroe Islands. Eight individuals, aged between 43 and 69 years old, participated in two semi-structured interviews. Thematic analysis revealed four main themes, including fear of consequences, starting exercise, hoping to keep on, and being social. According to the authors, these findings highlight the complexity of motivation, ranging from controlled forms (i.e., fear of complications) to more autonomous motivations (i.e., enjoyment, social connection). This study thus provides insights into the motivational factors influencing physical activity among individuals with T2D.

An interesting meta-analysis of randomized controlled trial conducted by Sun et al. examined the effects of Tai Chi on glycemic control in T2D. Meta-analytic results showed that Tai Chi significantly reduces fasting blood glucose, HbA1c, triglycerides, LDL-cholesterol, and some inflammatory markers such as hs-CRP, IL-6, and TNF-α. Subgroup analysis indicated optimal fasting glucose reduction with the standardized 24-form Tai Chi routine; interventions of 12 weeks or more; frequency greater than 5 sessions per week, and daily exercise duration equal to or greater than 60 min. These findings suggest that Tai Chi promotes benefits for T2D management.

Another bibliometric and visualization analysis by Jin et al. used CiteSpace to investigate the hotspots and development trends in exercise intervention mechanisms for T2D. According to the results, research on this Research Topic has focused on changes in biological effects and molecular mechanisms (e.g., improvement in pancreatic beta cell function, exerkines, and epigenetic mechanisms). Emerging research focuses on the heterogeneity of the response to exercise, circadian rhythm regulation, neurotrophic factors, transcription factors, and mitochondrial function. According to the authors, future research should explore the interactions between different mechanisms and elucidate the appropriate modes, dosages, and intensities of exercise for precise intervention in T2D.

Collectively, these studies highlight the importance of an integrated, multifactorial approach to the prevention and/or management of T2D, especially in the elderly population. Studies reinforce the important role of healthy lifestyle factors, including regular exercise and a balanced diet, in effectively managing the disease and reducing the risk of associated comorbidities. Furthermore, studies have focused on the association between T2D and cognitive function, which is often compromised in older adults. One study suggests that cognitive dysfunction could play a mediating role in the relationship between T2D and mortality in this population. Given the projected increase of the disease, it is necessary to develop more coordinated public health strategies focused on the elderly.
